# Improving students’ learning environment by DREEM: an educational experiment in an Iranian medical sciences university (2011–2016)

**DOI:** 10.1186/s12909-019-1839-9

**Published:** 2019-10-29

**Authors:** Hamid Bakhshialiabad, Golnaz Bakhshi, Zahra Hashemi, Amirhosein Bakhshi, Faroukh Abazari

**Affiliations:** 1 Department of Medical Education, Molecular medicine Research Center (mmrc) Faculty of medicine, Rafsanjan University of Medical Sciences, Rafsanjan, Iran; 2 Academic member of Educational development center, Rafsanjan University of Medical Sciences, Rafsanjan, Iran; 3 Molecular Medicine Research Center, Rafsanjan University of Medical Sciences, P.O. Box 771751-735, Rafsanjan, Iran; 4 Islamic Azad University, School of Medical Sciences, Baft, Iran; 5 Department of Medical sciences, Faculty of Medicine, Rafsanjan University of Medical Sciences, Rafsanjan, Iran; 6 Islamic Azad University, Yazd, Iran; 7 Department of health nursing, Kerman University of medical sciences, school of Nursing and Midwifery, Kerman, Iran

**Keywords:** Students, Learning environment, Educational programs, Rafsanjan University of Medical Science (RUMS)

## Abstract

**Background:**

Students’ perception of the educational setting is an important source for improving and applying changes to the educational environment. In this study, we reassessed undergraduate students’ perception of the educational environment at two colleges of RUMS-Iran in the academic years of 2011 and 2016.

**Methods:**

In the present prospective study, the DREEM inventory consisted of seven courses for undergraduate paramedical and nursing-midwifery students (*n* = 982). After the first stage, educational seminars and workshops were set up for academic members and faculty staff on deficiencies and the ideal climate for optimizing the educational environment. The results of students’ responses in 2011 were compared with those assessed in 2016. For the data analysis, the independent t-test and the one-way ANOVA were utilized.

**Results:**

In the academic year of 2010–2011, the DREEM inventory scored 115.33/200 (57.66%); it also scored 123.47/200 (60.7%) in the academic year of 2015–2016 (*p* ≤ 0.01). There was an interesting feeling about the first-year education, and female students felt a more positively perceived learning environment during all courses than male students at both stages of the study. There were significant positive differences (2 to 7%) in all domains of the components of DREEM in all courses between the academic year of 2010–2011 and the academic year of 2015–2016, showing that the DREEM score had changed and increased (*p* < 0.05), in the latter case.

**Conclusions:**

Positive differences were observed in DREEM scores between the two stages of the study. DREEM helped reduce the areas of deficiencies in students’ perception of many aspects of the educational environment. It also helped identify problematic areas in the improvement. In addition, DREEM could be used to optimize and make modifications to the educational environment.

## Background

Students’ perceptions of their learning environment are valuable in revising and refining the quality of the educational environment [1]. Educational research has shown that the significance of orientation towards learning is positively correlated with students’ perception of the educational environment [2]**.** An educational environment is composed of the three components of the physical environment, as well as the emotional and intellectual climates [1]. The educational climate refers to various physical sets, contexts, and values in which students receive education [3]. The educational environment affects positively the motivation, happiness, achievement, success, and satisfaction of students [4–6]. The quality of the educational environment indicates the efficiency of an educational program. The competency of the health care staff influences the safety and health of patients, and their education is essential to health initiatives [7, 8]. Based on the guidelines of the World Federation for Medical Education (WFME), improving the learning environment has been recognized as one of the objectives of the assessment of medical education programs [8–10]. Medical educators believe that clinical and theoretical environments exert significant impacts on the attitudes, knowledge, skills, progress, and behaviors of medical students [9–11]. If students’ perception in this context is considered as the basis for improvement, the measuring, implementing, modifying, and reforming of the educational environment will be possible [12]. Targeted learning is definitely associated with students’ perception of the educational environment, which influences their learning experiences and outcomes [12, 13]. Students’ perception of the educational environment has been studied by many educational institutes [4, 5, 12, 13].

In the latest attempts to boost quality assessment monitoring and guarantee the education of health professionals for the purpose of student-centered teaching and learning, a great deal of interest is observed in this field [6–8]. Students’ accomplishments can be influenced by their educational climate. The study of the learning environment is beneficial for the identification of students’ perceptions of their environment and support the staff to reflect on, plan for, and combine proper teaching approaches for the improvement of the educational environment.

The study of the educational environment deals with assessing what happens at school [13] and providing the available comprehensive evidence on the education process, the consequences of which being used to boost students’ satisfaction and achievements [4–6]. A key indicator of the quality of learning experiences is students’ satisfaction, being linked to some important variables. To provide high-quality and student-centered curricula, the assessment of the educational environment and the use of an appropriate tool are required [11]. Researchers in the field of education have tried to explain and measure the education environment [13–15], with the most widely used tool being the Dundee Ready Education Environment Measure (DREEM) [13]. DREEM is a validated and universal instrument [13], which has been translated into several languages, including the Persian, Swedish, Greek, Spanish, Chinese, Arabic, Malay, Portuguese, Norwegian, and Thai languages [6, 13–18]. DREEM has been recognized as a reliable tool in a series of medical settings, by which administrators can detect limits and consider alterations to curricula [5, 19, 20].

DREEM has so far been used to identify weaknesses in curricula [6, 21, 22] and determine the impact of new curricular interventions [22–24]. It is also used for recognizing the distance between students’ anticipations and experience [25], as well as students’ understandings in different conditions at medical schools [13, 26].

Students’ perception of their medical education [27, 28] at different stages has also been examined by DREEM [5, 7, 29]. Furthermore, this valuable tool has been used in undergraduate courses by health professionals and in health care fields universally [3, 30], including medicine, dentistry, nursing, midwifery, anesthesiology, medical emergencies, paramedical sciences, and chiropractic learning environments [5, 25, 31, 32]. A significant part of identifying the efficiencies and fields where developments could be made is to receive ‘feedbacks’ from students on designing the learning environment [18].

The Dundee Ready Educational Environment Measure (DREEM) instrument is commonly used toا evaluate the learning environment of medical sciences as well as other health sciences in various academic settings, with the results being used to compare different institutions offering health courses. Since its development about 10 years ago, this instrument has been used to outline the strengths and weaknesses of institutions based on students’ perceptions. DREEM, in IRAN, is not commonly utilized to determine students’ perceptions of their learning environment. Aside from the lack of records on the strengths or weaknesses of institutions offering health courses, the Commission on Higher Education of the Ministry of Health and Medical Education has also indicated the need for some reforms in the schools of medical sciences in Iran. Based on this requirement, they must work aimed at educating graduates, by emphasizing competence-based learning, eliminating problems in the educational environment, and also improving the educational methodology, behavior, and professional ethics of instructors at school. In recent years, novel instruction methodologies, such as the use of technology, have been adopted and used at some higher education institutions in Iran, yet a gap exists between the prioritization level of this educational reform and its level of use. Hence, to initiate relevant educational reforms, it is required that the health science students’ perceptions of their learning environment be assessed In this respect, in this study, identify problems, design training programs, and determine the impact of these educational intervention programs on changing students’ perceptions and compare results of before and after interventions based on age, gender and year of enrolment using the Dundee Ready Educational Environment Measure (DREEM). This study was carried out at Rafsanjan University of Medical Sciences (RUMS; undergraduate faculties This survey was also conducted to assess and compare the improvement of undergraduate student’s perceptions that studying in nursing-midwifery, and paramedical faculties, of their learning environment at two stages, i.e. before and after educational interventions, among academic members and staff at RUMS (2011 and 2016). This educational intervention model was used to identify problem areas to be considered and in the future for improvement and to apply reforms to learning environments to improve them.

## Methods

### Study design, samples, setting, and ethical considerations

This prospective quasi-experimental study was designed and implemented before and after interventions, using a standardized self-report scale. Data collection was done at two stages and at a five-year interval (2011 vs. 2016). The Board of Research Ethics at RUMS approved the study. The samples collected included 982 students at both stages. The study subjects were selected from the total population of students (first stage 610 and second stage 628 students) by a stratified random sampling method based on educational courses, sex and entry year of students. The participants at every stage of the survey included undergraduate health science students in the fields of nursing [*n* = 202], midwifery [*n* = 64], radiology [*n* = 51], operating room nursing [*n* = 46], laboratory sciences [*n* = 44], medical emergencies [*n* = 35], and anesthesia [*n* = 51]). The students received instructions on the study and were informed that all data collected would be kept confidential. Next, written informed consent forms were obtained from the subjects. To collect data [12, 18, 28, 33] on the students’ demographic characteristics, including gender, year of admission, and age, the Persian version of DREEM was used, with its validity and reliability approved. The sampling process continued to complete the Sample size required and replace the incomplete cases.

According to the results of the first stage of the study, DREEM items with poor perception (mean score ≤ 2) were extracted and quantified (Table 1) In addition, students evaluated the educational environment to have a lot of problems. A total of 38 items had aspects of the learning environment that could be enhanced (mean score ≤ 3). The students’ most difficult problems were observed in the fields of the faculty atmosphere, learning, teachers, their teaching methods, and social self-perception. In the meantime, the research team planned and ran training programs based on the problems. Educational programs included 12 short-term educational programs within a year in the fields of communications, teaching and assessment methods and materials, educational environment, self-study methods, and learning styles in the forms of workshops, conferences, and panels for teachers and staff, with trainings provided during non-administrative hours.

**Table 1:** Problematic Items (mean score ≤ 2) in subscales of Learning environment before interventions (2011)

I: students’ perceptions of learning 23. The atmosphere is relaxed during lectures 24. The teaching time is put to good use 37. The teachers give clear examples 12. The school is well-timetabled 7. The teaching is often stimulating	IV: students’ perceptions of atmosphere 32. The teachers provide constructive criticism here 13. The teaching is student-centered
II: students’ perceptions of teachers 50. The students irritate the teachers 8. The teachers ridicule the student 9. The teachers are authoritarian 48. The teaching is too teacher-centered 35. I find the experience disappointing 25. The teaching over-emphasizes factual learning	V: students’ academic self-perceptions 28. I seldom feel lonely 46. My accommodation is pleasant 17. Cheating is a problem in this school
III: students’ social self-perceptions 3. There is a good support system for students who get stressed 6. The teachers are patient with patients 14. I am rarely bored on this course	

The data were collected from the undergraduates of two school students, including paramedical students and nursing-midwifery students, using the DREEM inventory at RUMS, Iran. After the first stage of the survey based on deficiencies and the ideal educational climate, some educational workshops and seminars (*n* = 12), were planned and run for academic members and faculty staff by the educational development center (EDC). Next, the second stage was passed through for the latter five-year period, with its results compared with those of previous assessments (2011).

The DREEM scale is composed of 50 items, each of which scored on a five-point Likert scale (4 = strongly agree, 3 = agree, 2 = unsure, 1 = disagree, and 0 = strongly disagree) with the maximum score of DREEM being 200. However, nine out of 50 items (i.e. numbers 4, 8, 9, 17, 25, 35, 39, 48, and 50) were negative statements and had to be recorded reversely.

The questionnaire assigns an overall ‘score’ to the course. The educational environment was divided into five major areas, including the perception of learning (12 items, max score of 48), the perception of teachers (11 items, max score of 44), academic self-perception (8 items, max score of 32), the perception of atmosphere (12 items, max score of 48), and social self-perception (7 items, max score of 28).

The data analysis was performed using SPSS software (version17; IBM, Armonk, NY, USA). Continuous variables were summarized as the mean and the standard deviation (SD), with the independent *t*-test and the one-way ANOVA used for the analysis. In this study, the significance level was set at *P* ≤ 0.05.

## Results

At the first (2011) and second (2016) stages of the survey, a total of 986 usable responses were obtained (response rate in first and second stages were 95 and 94%), with 493 questionnaires at every stage consisted of 201 (42%) males and 292 (58%) female students. The ages ranged from 17 to 30, with the mean age of 22 ± 5. In this context, 35.4% (175) of students were in first year, 23.5% (116) of them were in second year, 24.5% (123) of them were in the third year, and 16.5% (81) of them were in fourth year of education. There was no statistically significant difference in demographic characteristics between the students at both stages.

Analyzing of DREEM items in first stage of survey (2011) recognized that nineteen items had mean scores of less than two of five, with a usual of two to six items in each domain. Some items scored reliably badly indicating cause for concern, such as, lack of a support system for stressed students, and school time-tabling, authoritarian teachers and feedback from teachers and memorization of facts. The maximum mean score was 3.1 (I am encouraged to participate in class). A total of 38 items had aspects of the learning environment that could be enhanced, in the other hand, the items were with mean score less than 3 of 5.In addition, students evaluated the educational environment to have a lot of problems. DREEM items with poor perception of students were noted separately (Table 1). Based on the problems previously reported by the students, the training programs needed to address and enhance students’ understanding were designed.

At the first stage of the survey (2011), the total mean score was 113.5 (SD 21.9) out of the maximum of 200, being equal to 56.74% of the maximum score (95% confidence interval [CI]: 110–118); in addition, at the second stage (2016), the scores were 123.4 (SD16) and 61.74% [CI 95%: 122–124]. These overall scores of DREEM show an increase (5%) in the first stage compared to the second stage of the survey.

A statistically significant difference was observed between the subscale (*p* = 0.01) and the total DREEM scores (in five areas) (*p* = 0.001) at the two stages (Table 2).

**Table 2:** Mean (SD)[Percentage] and differences of subscale and total DREEM scores for seven courses of Rafsanjan University of Medical sciences (RUMS) students at 2011 and 2016

Domain	Stages of survey		Interpreting^§^ DREEM Score	Mean difference	*P*.value
	1st stage [2011] (*N* = 493)	2secd stage [2016] (N = 493)			
Students perception of learning (SPL)	27.1 (5.9) [57.2%]	29.5 (3.7) [61.58%]	A more positive perception	2.44 (4.5)*	0.001
Students Perception of teachers (SPT)	24.8 (5.6) [55.9%]	25.3 (3.8) [57.57%]	Moving in the right direction	0.49 (3.2)*	0.001
Students’ Academic self -Perception (SASP)	20.1 (4.7) [64.11%]	24.7 (4.07) [77.1%]	Feeling more on the positive side	4.6 (2.8)*	0.001
Students Perception of Atmosphere (SPA)	27.1 (5.6) [55.89%]	28.1 (5.5) [58.6%]	A more positive atmosphere	0.97 (1.1)*	0.001
Students Social Self- Perception (SSSP)	15.4 (4.9) [56.38%]	17.8 (4.4) [63.7%]	Not too bad	2.35 (2.6)*	0.001
Total DREEM item score for the group	113.5 (17.3) [56.74%]	123.48 (16) [61.74%]	More positive than negative	8 (8)*	0.001

*Statistical significance difference. ^**§**^ McAleer and Roff (2001)

The mean difference in the subscale group between the two stages was statistically significant, and the total DREEM and subscales were interpreted based on the study by McAleer and Roff (2001), which were in a ‘more positive than negative’ educational environment, indicating no alteration in the category of the two stages. Hence, the subscale of SPL was in the category of ‘a more positive perception’, the subscale of SPT was in the category of ‘moving in the right direction’, the subscale of SASP was in the category of ‘feeling more on the positive side’, the subscale of SPA was in the category of ‘a more positive atmosphere’, and the subscale of SASP was in the category of ‘not too bad’. In addition, there was a significant positive difference in all subscales between the two stages of the study (Table 2).

The mean difference in the subscale group (five areas) at the second stage (2016) of the survey was higher than that of the first stage (2011) [113.5(SD 17.3) vs. 123.48(SD16)], with the 95% confidence interval of the difference having been 8.07 to 9 (*p* = 0.001). The overall score indicates that participants perceive the educational environment (EE) more positively than negatively at both stages. The highest mean differences between the two stages were observed in students’ academic self-perception (SASP), students’ perception of learning (SPL), and students’ social self-perception (SSSP). Students’ perception of teachers (SPT) and students’ perception of atmosphere (SPA) yielded the lowest mean differences, respectively. In addition, there was a statistically significant correlation [at the level of 0.01 (2-tailed)] between the five areas of DREEM at the first and second stages.

At both stages, the mean scores of the female students were found out to be higher than those of the male students. The overall DREEM scores showed significant differences between the male [116.2, 58.1% V 14.16, 62.8%] and female [110.7, 55.36% vs. 122.4, 61.2%] students at the first (2011) and second (2016) stages of the survey (Table 3).

**Table 3:** Mean (SD) of subscales and total DREEM scores for Rafsanjan University of Medical Sciences by disciplines at 2011 and 2016

Health science Discipline	SPL (max. Score = 48)		SPT (max. Score = 44)		SAP (max. Score = 38)		SPA (max. Score = 48)		SSP Max. score = 28)		Total DREEM Score (max. Score = 200)		Percentage for DREEM	
	2011	2016	2011	2016	2011	2016	2011	2016	2011	2016	2011	2016	2011	2016
Nursing *n* = 202	27.4 (5.9)	30.27 3.98	24.3 (5.9)	25.97 3.86	20.3 (4.5)	24.96 4.05	26.8 (5.6)	28.85 5.8	15.6 (4.2)	19.26 3.79	114.3 (20.6)	126.95 (17.8)	57.15	63.49
Midwifery *n* = 64	24.8 (4.2)	28.42 2.96	23.5 (4.2)	24.48 2.98	18.3 (5.1)	23.39 3.82	25.4 (6.6)	26.51 6.7	15.2 (4.7)	16.8 4.36	106.23 (22.7)	118.33 (15.37)	53.1	59.16
Laboratory sciences *n* = 44	28.6 (5.8)	30.98 3.65	25.5 (8.4)	25.87 4.8	22.2 (6.5)	26.6 4.27	28.2 (5.6)	29.35 5.74	14.1 (4.8)	18.18 4.48	120.95 (21.7)	129.29 (16.88)	60.2	64.64
Anesthesia *N* = 51	27.8 (3.3)	28.7 3.51	24.4 (3.4)	23.81 3.43	21.7 (5.2)	23.41 3.93	28.9 (3.1)	27.36 3.97	16.3 (10.8)	16.9 7.59	120.58 (14.3)	118.21 (15.95)	60.3	59.1
Radiology *n* = 51	24.9 (5.1)	29.18 3.35	23.2 (8.7)	25.56 3.83	18.5 (3.3)	23.58 3.56	24.1 (5.6)	25.49 4.43	14.7 (3.3)	14.94 3.47	105.6 (19.5)	116.56 (11.36)	52.3	58.28
Operation room nursing *n* = 46	29.6 (7.9)	28.24 3.54	29.8 (3.7)	25.38 3.43	21.3 (3.5)	25.06 (3.77)	29.5 (11.2)	29.46 (5.06)	14.5 (4.3)	16.76 2.42	121.87 (21.7)	122.64 (12.21)	60.9	61.32
*Medical emergency* *n* = 35	28.8 (4.6)	29.36 (3.38)	23.9 (4.1)	24.28 (3.43)	22.7 (2)	26.39 (4.01)	25.8 (3)	28.55 (3.51)	16.6 (2.1)	18.07 (1.97)	117.8 (11.7)	124.64 (11.85)	58.9	62.19
Total Mean *N* = 493	27.4	29.56	24.6	25.33	20.5	24.71	26.7	28.13	15.7	17.83	113.5	123.48	56.7	61.74
*P-*value	0.03	0.001	0.4	0.001	0.001	0.001	0.001	0.001	0.001	.001	0.001	0.001	0.001	0.001
Lower 95% CI	26.15	29.47	23.5	25	19.5	24.37	26.12	27.65	14.67	17.45	110.62	124.66	55.67	122.12
Upper 95% CI	27.3	29.88	24.6	25.65	20.4	25.6	27.28	28.6	15.51	18.21	114.71	129.24	57.03	124.85
Mean Percentage for each Domain	57.2	61.5	55.9	57.5	53.9	77.7	55.8	58.6	56.0	63.7	56.7	61.74		

Abbreviations: CI, confidence interval; SD, standard deviation; DREEM, Dundee Ready Education Environment Measure; SAP, students’ academic self-perception; SPA, students’ perception of Atmosphere; SPL, students’ perceptions of learning; SPT, students’ perceptions of teaching; SSP, students’ social self-perception

Table 3 shows the DREEM scores of various areas for the two stages of the survey for the courses. The highest differences in the overall DREEM scores were seen among the students of midwifery, medical emergency, and radiology. In contrast, the lowest differences in the total DREEM scores were observed among the fields of laboratory sciences and operative room nursing. In addition, the results showed that the total score of the students of anesthesia had decreased at the second stage. Furthermore, the differences between the two stages were significant in all courses (*P* = 0.001).

Moreover, the highest mean score differences between the two stages were seen in the fields of academic self-perception (SASP), social self-perception (SSP), and perception of learning (SPL) in all courses, with statistically significant differences (Table 4).

**Table 4:** Mean (%) of subscale and total DREEM scores for Rafsanjan University of Medical Sciences students by gender at 2011 and 2016

Subscale	Female		Male		Total		*P*-value
	2011	2016	2011	2016	2011	*2016*	
Perception of learning (max =48)	27.38 (57.05)	29.69 (61.85)	27.58 (57.47)	29.35 61.16	27.4 (57.2)	*29.58* (61.58)	0.8
Perceptions of teachers (max =44)	24.62 (55.96)	25.19 (52.26)	24.56 (55.83)	25.54 58.05	24.6 (55.9)	25.33 *(57.57)*	0.9
Academic self-perception (max = 38)	20.7 (64.73)	24.74 (78.13)	20.13 (62.92)	24.67 (77.26)	20.5 (64.11)	24.71 (77.79)	0.3
Perceptions of atmosphere (max =48)	6.9 (56.04)	28.29 (58.95)	26.68 (55.59)	27.86 (58.06)	26.8 (55.89)	28.13 (58.6)	0.7
Social self-perception (max =28)	16.16 (57.74)	18.22 (65.07)	15.03 (53.71)	17.23 (61.54)	15.7 (56.38)	17.83 (63.7)	0.09
Total DREEM	116.2 (58.1)	124.16 (62.08)	110.72 (55.36)	122.41 (61.20)	113.5 (56.74)	123.48 (61.78)	0.03
p.value	0.001		0.001		0.001		

Abbreviation: DREEM, Dundee Ready Education Environment Measure

However, some statistically significant differences were observed between the mean scores of the DREEM fields and the total DREEM at the two stages of the survey among the first, second, third, and fourth-year students (*P* = 0.03). The total scores of the differences between the first and second stages of the survey in the second and third year students were significantly higher than those of other students (Table 5).

**Table 5:** Mean (Percentage) of subscale and total DREEM scores for Rafsanjan University Medical Sciences students by year of enrolment at 2011 and 2015

Subscale	1st year		2nd year		3rd year		4th year		Total		*P*-value
	2011	2016	2011	2016	2011	2016	2011	2016	2011	*2016*	
Perception of learning (max = 48)	28.39 (59.1)	*29.4* *(61.2.)*	27.06 (56.4)	28.13 (58.6.)	26.16 (54.3)	30.75 (64.1)	28.97 (60.3)	29.84 (62.2)	27.4 (57.2)	29.56 (61.6)	… ..
Perception of teachers (max 44)	26.49 (60.2)	26.1 (59.3)	23.23 (52.8)	23.86 (54.2)	23.21 (52.7)	25.07 (56.9)	25.38 (57.7)	26.39 (59.9)	24.60 (55.9)	25.33 (57.6)	0.001
Academic self-perception (max =38)	20.88 (65.2)	24.71 (77.4)	20.8 (65.0)	23.97 (74.0)	19.8 (61.9)	25.96 (80.9)	20.63 (64.5)	24.37 (78.5)	20.5 (64.1)	24.71 (77.8)	0.48
Perceptions of atmosphere (max =48)	28.29 (58.94)	28.85 (60.1)	25.69 (53.5)	26.94 (55.5)	25.9 (53.9)	29.15 (60.7)	27.19 (56.6)	28.28 (58.9)	26.8 (55.8)	28.13 (58.6)	0.008
Social self-perception (max =28)	15.66 (55.95)	17.79 (63.5)	15.68 (56)	17.78 (63.5)	16.19 (57.8)	18.65 (66.6)	15.3 (54.6)	17.28 (61.7)	15.7 (56.1)	17.83 (63.7)	0.8
Total DREEM	119.7 (56.86)	124.65 (62.38)	112.49 (56.24)	118.14 (59.0)	111.19 (55.59)	127.62 (63.81)	117.5 (58.75)	124.17 (62.08)	113.5 (56.74)	123.48 (61.74)	0.03

Abbreviations: DREEM, Dundee Ready Education Environment Measure; SD, standard deviation

## Discussion

The assessment of the educational environment is a vital part of program assessments [10, 34]. In this study, the DREEM scale was used to compare the perceptions of students in the fields of midwifery, nursing, radiology, operating room nursing, laboratory sciences, medical emergencies, and anesthesia of their educational environment, at RUMS in the two time periods of 2011 and 2016.

The overall mean DREEM score of the students of the first and second stage of the survey was found to have improved, but the mean DREEM scores at both stages were within the range of 101–150, indicating a ‘more positive than negative’ perception of the educational environment [10, 30]. Although this improvement is considerable after five-year quantitative and qualitative interventions in RUMS, there is an opportunity for obtaining maximum scores using positive interventions to promote the educational environment. The significant differences between the two stages of the study imply that perceived factors, such as the curriculum, structure, focus, and goals have been different among students in different time periods within the time span of the study. There was an increase in the scores of the five fields of DREEM, with the greatest progress observed in the students’ academic self–perception (SASP) and students’ social self-perception (SSSP). The increase in the scores indicate that some emotional factors affect the educational environment, which of course need further examination. The comparison of the mean scores, i.e. in total and the five subscales, showed that the values were higher in 2016 than in 2011.

Although the students of different majors and courses were exposed to similar curricula, academic requirements, teaching methods, and socio-demographic characteristics, the mean scores of the total DREEM and its five subscales varied for different majors and academic years in the students at both stages of the survey (2011 vs. 2016). Past studies reported findings consistent with the results of this study on the academic year that reported a better EE. Most studies reported that freshmen performed better either in terms of the total DREEM scores or some of its subscales than the seniors [12, 35–37]. However, opposite results have been reported by studies in Saudi Arabia and Philippines [13]^.^

The students of the paramedical college showed higher mean difference scores than those of nursing and midwifery colleges from the total scores. The differences in the total DREEM scores could be attributed to a number of factors associated with differences in the curriculum, faculty profile, subjects offered, types of academic requirements, educational programs, teaching methods, as well as socio-demographic characteristics of the students of the study. Notwithstanding the fact that traditional didactic courses are still run in the two colleges studied, the paramedical college accounted for higher mean scores than the nursing-midwifery college from the total score, with this reflecting the changeability of the EE.

The students of nursing, midwifery, laboratory sciences, and medical emergencies had higher mean scores of differences between the first and second stages, but the differences among the students of anesthesia, radiology, and the operating room were not considerable. The total DREEM scores of a nursing school in China [30], medical schools in Sri Lanka, Nepal, Nigeria, Saudi Arabia, the UK (Birmingham), Chile, Kuwait, Sweden, Jamaica, Trinidad, the dental school of Malaysia, the International Medical University (Malaysia) [37], the University of British Columbia’s Medical School [5], medical students of India [38] and Australia [36], the International University of Management (Bachelor of Nursing) [35], Indonesian nursing students [39], and similar studies [16, 21, 22, 40–44] were within the same score range of 101–150. According to DREEM interpretation guides, these scores are considered to be more positive than negative. There are also a few studies reporting an overall mean DREEM score of 130, such as the study on a Malaysian private nursing college [45], the study on a Chinese nursing school [46], a series of UK studies [24], and the study on Monash University in Australia [36].

The results indicated that the nursing-midwifery and paramedical schools of RUMS had a ‘more positive than negative’ status, having been only one level lower than the highest category of reachable scores. In addition, the students of innovative curricula were inclined to show more satisfaction with their educational environment than the students of more traditional curricula. According to the results, higher DREEM scores are more likely to be indicative of more student-centered curricula, while DREEM scores for conventional curricula are generally less than 120 out of 200 [11, 12, 30].

At both stages of the survey, the subscale scores of the DREEM indicated that the students’ perceptions of the learning environment were ‘positive’, and their perceptions of the teachers were ‘moving in the right direction’. The students’ perceptions of the atmosphere was ‘a more positive one’, and the students’ social self-perception was ‘not too bad’, whereas their academic self-perception was found out to be ‘more positive’. The sample’s mean perceptions were expressed in percentages, having been within the range of 53.44–56.87% for the five fields. These mean scores indicate that there is an opportunity for an improvement in the features measured by DREEM at schools. These findings are comparable with similar DREEM studies [16, 21, 22, 40, 41, 47, 48]. Students’ perceptions can be used to start making future changes and improvements. Medical education costs are high, and academic failures could be a great waste of resources of both the society and individuals [28]. Therefore, we are required to ensure that the learning environment is as encouraging as possible and ultimately try to reduce the risk of academic underachievement.

The significant differences between female and male students at both stages suggest that perceived factors, such as the curriculum, structure, emphasis, and goals are different for female and male students. There was a statistically significant difference between the genders in the overall DREEM scores; in addition, in terms of the individual subscales, academic, learning, and social perceptions were the areas of the highest difference between the two genders of the two stages, being comparable to other studies that showed female students’ perceptions were more positive [21, 30, 36, 49–52]. The results of the present study are significantly different from the studies that reported no significant gender difference or opposite results between females and males [20, 30, 43, 44, 47, 53]. Concerning gender differences, both genders couldn’t perceive their courses identically and had different learning styles [35, 52]. This variation in the total scores and domain scores may have been related to the gender profile of the respondents and other factors, such as different types of curricula [54–56], faculty profiles, and goals [28].

The mean scores of the DREEM and its five subscales varied based on the academic year at both stages of the survey (2011 vs. 2016). All students were exposed to the same curricula, academic requirements, and teaching methods with similar socio-demographic characteristics. The students’ perceptions of learning, atmosphere, and teachers varied according to the students’ academic year and level of education. According to the results of the present study, freshmen had the highest mean scores than sophomores, juniors, and seniors. In comparison to the second stage in which the mean scores were higher than the first one, the differences were similar to the results of other studies that reported reduced scores in seniors [35, 57]. This trend could have been caused by the learning environment and the fact that students become mentally tired of their being students, so they look forward to leaving their education life. In addition, the dissatisfaction that appeared in the form of the novelty of joining a body of health science students may have worn off, upon the start of the course [18, 57]. However, the difference does not follow a constant pattern from an academic year to another in other studies [22, 36, 58]. Further studies are required to be done on every single course to help make necessary alterations.

While the present study offered a profound insight into the learning environment as perceived by RUMS undergraduate students, conducting similar studies at other universities and at international levels seems to be plausible. In this context, there were some limitations, with one of which having been that individual items were not analyzed, and qualitative data were not collected to deal with specific problems or highlight the strengths of the university or various courses more deeply. The paramedical college had higher mean difference scores of the total score than the nursing-midwifery college in 2011 and 2016. The differences in the total DREEM scores may have been attributed to several factors, including the curriculum, faculty profile, subjects offered, types of academic requirements, different educational programs, teaching methods, as well as socio-demographic characteristics of the students studied. Medical science students require great academic and professional skills. One of the practical ways of assessing the quality of education in medical sciences is to examine students’ opinions. Unlike single-step studies, the present study was done by recognizing problems at the first stage and planning training courses to improve the learning environment. Although interventions were partially effective, student education could not be affected positively by them. The design of the before and after study can also be considered as a limitation of this study. According to the researcher’s experience, to eliminate the limitations, and a similar study must be done in just one field of the study, selection of control groups and during two semesters to determine the definite impact of training interventions.

## Conclusions

The results of the present study indicate that the students enrolled at RUMS generally have had positive perceptions of their education environment at both stages of the study, i.e. 2011 and 2016. Better perceptions were reported in the second stage of the study than in the five years earlier (2011) in females. In addition, dissimilarities among academic levels were consistent with the results of other studies in this field. These issues as well as the differences between courses and study pathways need more examination by analyzing specific items and sub-cohorts.

DREEM provides a clear indication of the priorities required for reforming curricula. These results could be used as the basis for the longitudinal quality assessment of students’ perceptions at RUMS schools. Improving the quality of the educational environment and the effectiveness of educational programs are also necessary in educating students, thereby enhancing their learning capacity, increasing their interest in learning, inspiring them, producing better learning outcomes, promoting academic developments, and elevating the sense of well-being.

## Data Availability

The datasets used and analyzed during the current study are available from the corresponding author on reasonable request.

## Abbreviations

DREEM: Dundee Ready Education Environment Measure
RUMS: Rafsanjan University of Medical Sciences
